# Prospective Use of High-Refractive Index Materials for Single Molecule Detection in Flow Cytometry

**DOI:** 10.3390/s18082461

**Published:** 2018-08-01

**Authors:** Joshua A. Welsh, Julia Kepley, Ariel Rosner, Peter Horak, Jay A. Berzofsky, Jennifer C. Jones

**Affiliations:** 1 Vaccine Branch, Center for Cancer Research, National Institutes of Health, Bethesda, MD 20892, USA; joshua.welsh@nih.gov (J.A.W.); julia.kepley@nih.gov (J.K.); arijrosner@gmail.com (A.R.); berzofsj@mail.nih.gov (J.A.B.); 2 Optoelectronics Research Centre, University of Southampton, Southampton SO17 1BJ, UK; peh@orc.soton.ac.uk

**Keywords:** extracellular vesicles, flow cytometry, light scatter, scatter modelling, complex refractive index, scatter labels, NanoTags, single-epitope detection

## Abstract

Phenotyping extracellular vesicles (EVs), where surface receptor expression is often as low as one molecule per EV, remains problematic due to the inability of commercial flow cytometers to provide single-fluorescent molecule sensitivity. While EVs are widely considered to be of great potential as diagnostic, prognostic and theranostic biomarkers, their use is currently hindered by the lack of tools available to accurately and reproducibly enumerate and phenotype them. Herein, we propose a new class of labels that leverage the biophysical properties of materials with unique complex refractive indices and demonstrate that this class of labels has the possibility of allowing single-epitope detection using conventional flow cytometry.

## 1. Introduction

Extracellular vesicles (EVs) are small (30–1000 nm) membranous vesicles with the majority being <150 nm in diameter [1]. Due to their small surface area, when compared to cells, the epitope expression of the majority of EV surface markers is below the detectable range of conventional high throughput, multi-parameter detection techniques, such as flow cytometry [2].

Conventional flow cytometry (cFC) is capable of distinguishing thousands, to a few hundred, fluorescent molecules. The surface expression of highly abundant lymphocyte surface marker, such as CD14, is in the region of 100,000 copies per lymphocyte. If this epitope density were scaled to a 100 nm EV, it would equate to <30 CD14 copies per vesicle [3]. Therefore, an unmet need in the EV field is the development of labels that enable single-epitope detection on single EVs, ideally utilizing conventional detection equipment.

cFC quantifies the expression of proteins using fluorescent probes, which tend to be in the form of fluorescently conjugated antibodies. While these have proven to be a powerful tool for cellular analysis in the immunology field, in their current form they are insufficient to quantify EV surface protein expression on the majority of available flow cytometers. While newer generations of fluorescent labels have emerged, such as QDots, they remain generally inadequate for low surface epitope quantification. The use of QDots as labels for time-limited detection of individual targets is also complicated by stochastic optical fluctuation, commonly referred to as “blinking” [4].

When the objective of an analysis is to enumerate the number of EVs which are positive for a specific marker in a sample, the method of enumeration must be able to detect each EV with one or more specific surface marker. In this case, it is essential to use labels and instruments capable of detecting single labels, with single label sensitivity. On the other hand, when the objective is to quantify or compare how many receptors are present on individual EVs, there must be a constant label-to-target ratio and labels with monovalent target binding-sites are needed for single molecule detection and surface receptor quantification on EVs. Other standardization steps should also be considered when enumerating EVs, such as dilution controls, MESF beads, and/or scatter modelling [5]. Most commercially available techniques utilizing QDots use these labels in polyvalent forms but new avenues have emerged that have attempted to create monovalent QDots which may be directly transferrable to the conjugation of other nanoparticles [6,7,8].

Here, we propose the development of a novel class of nanoscale molecular tags (NanoTags) that could enable single label and therefore single molecule, detection using conventional flow cytometers. These NanoTags would be composed of materials with high refractive indices and/or high optical absorption and unique spectral scattering properties that would enable both low epitope number enumeration and spectral phenotyping of small particles, such as extracellular vesicles.

In this paper, we shall demonstrate how NanoTag compositions may be selected for unambiguous optical detection and how multiple NanoTag compositions could be used simultaneously to allow particle phenotyping and still be distinguished from one another. Our analysis is based on numerical modelling of wavelength dependent light scattering by nanoparticles and comparison with flow cytometry data.

## 2. Materials and Methods

### 2.1. Particle Refractive Index

To first identify particles with useful optical properties that would aid increased light scattering, the refractive index and extinction coefficients were collated, Supplementary Figure S1. Particle refractive indices and extinction coefficients across wavelengths 300–800 nm were compiled from the literature for the following compositions: gold, silver, iron oxide, titanium dioxide, copper, platinum, lead and zirconium [9,10,11,12,13]. The refractive indices of polystyrene and water were calculated at each wavelength using the corresponding Sellmeier equations [14,15].

The compositions selected were narrowed down by with the inclusion criteria of being currently sold in a nanoparticle format and having a high refractive index (e.g., Titanium Dioxide) or extinction efficient (e.g., Lead) or having a medium refractive index and extinction coefficient (e.g., Gold, Silver, Copper).

### 2.2. Particle Analysis Using Flow Cytometry

Flow cytometry measurements were performed on a MoFlo Astrios EQ (Beckman Coulter Life Sciences, Indianapolis, IN, USA) and FACS Symphony (BD Life Sciences, Franklin Lakes, NJ, USA) flow cytometers. The Astrios EQ is a jet-in-air system with 5 lasers (355, 405, 488, 561 and 640 nm wavelength), with side scatter collection (SSC) detection possible at 405, 488, 561 and 640 nm. Detailed description of Astrios EQ setup and detection thresholds can be found in the literature [16]. The FACS Symphony is a cell-analyzer with 5 lasers (355, 405, 488, 532 and 640 nm) with SSC detection only possible at 488 nm with the default device configuration. The equipment parameters for particle enumeration via SSC were set to a triggering threshold of 200, a voltage of 350 and a low fluidics rate.

For measurements of scattered power versus diameter, NIST-traceable polystyrene beads (100, 125, 147, 203, 296, 400, 600, 799, 994 nm diameter) (Thermo Fisher Scientific, gift from E. van der Pol, Waltham, MA, USA) and silica beads (182, 315, 359, 405, 548, 800, 1000 nm) (Kisker Biotech, gift from E. van der Pol, Amsterdam, The Netherlands) were analyzed at a concentration of 0.1–1 × 10^8^ particles per mL and confirmed the fit of predicted scatter versus acquired scatter using previously published flow cytometer methodology [17].

For high-refractive index particle acquisition modelling comparisons, Ag particles, 20, 40, 60, 80 and 100 nm in diameter (Cytodiagnostics, Burlington, ON, Canada), Au particles, 20, 40, 60 and 80 nm in diameter (Cytodiagnostics) and 100 nm polystyrene NIST-traceable beads (Thermo Fisher Scientific) and 200 nm fluorescent polystyrene beads (Thermo Fisher Scientific) were diluted to a concentration between 0.1–1 × 10^7^ particles per mL before acquisition on each instrument. Linear dilutions of 40, 60, 80 nm Au particles were performed to confirm working cytometer acquisition concentration that maintained single-particle detection (Supplementary Figure S2). Particle concentrations were determined using manufacturer percentage solids. Spike-in particle concentrations were determined using nanoparticle tracking analysis.

Data was acquired using the acquisition software Summit v6 on the Astrios EQ and Diva v8 for the FACS Symphony. Upon completion of data acquisition, files were exported from the acquisition software and imported into FlowJo v10 (TreeStar) for post-acquisition analysis. Flow cytometry files can be found at: https://flowrepository.org/id/FR-FCM-ZYL7 and https://flowrepository.org/id/FR-FCM-ZYL6.

### 2.3. Flow Cytometer and Particle Spectral Scatter Modelling

All numerical modelling was performed with MATLAB v9.3.0 (The MathWorks Inc., Natick, MA, USA). The cumulative power of light scattered by a spherical particle of fixed diameter (20, 40, 60 80, 100 nm), reaching a detector was calculated using Mie theory and implemented numerically with scripts that built upon those from Matzler [18]. The calculations used resemble those of van der Pol et al. [14] and Fattaccioli et al. [15]. This software is available at: http://www.joshuawelsh.co.uk/scatter-diameter-software/. Particle light scatter modelling focused on the side scatter, that is, on light collected perpendicular to the direction of illumination. The side scatter collection optics of the Astrios EQ flow cytometer have a circular collection aperture with a limiting half-angle approximated to be 29° [17,19]. The particle suspension medium was modelled using the optical properties of water. Fitting of the modelled data to the experimental data, in order to produce curves of scattered power (or equivalently, scattering cross section) versus particle diameter, was carried out using the method of van der Pol et al. [14] using a single scale factor to convert between simulated scattering cross sections and experimentally measured power levels. Flow cytometry data was converted from arbitrary units to scattering cross-section in nm^2^ using linear regression of predicted versus acquired values from modelling (Supplementary Figure S3).

### 2.4. Particle Analysis Using Nanoparticle Tracking Analysis (NTA)

Gold and silver particle diameter distributions and concentrations were measured using NTA with a NanoSight LM10 instrument (Malvern, UK), equipped with a 405 nm LM12 module and EMCCD camera (DL-658-OEM-630, Andor, Belfast, UK). Video acquisition was performed with NTA software v3.2, using a camera level of 14. Three 30 s videos were captured per sample. Post-acquisition video analysis used the following settings: minimum track length = 5, detection threshold = 4, automatic blur size = 2-pass, maximum jump size = 12.0.

## 3. Results

### 3.1. Spectral Scatter Modelling of Different Particle Compositions

**Figure 1:**
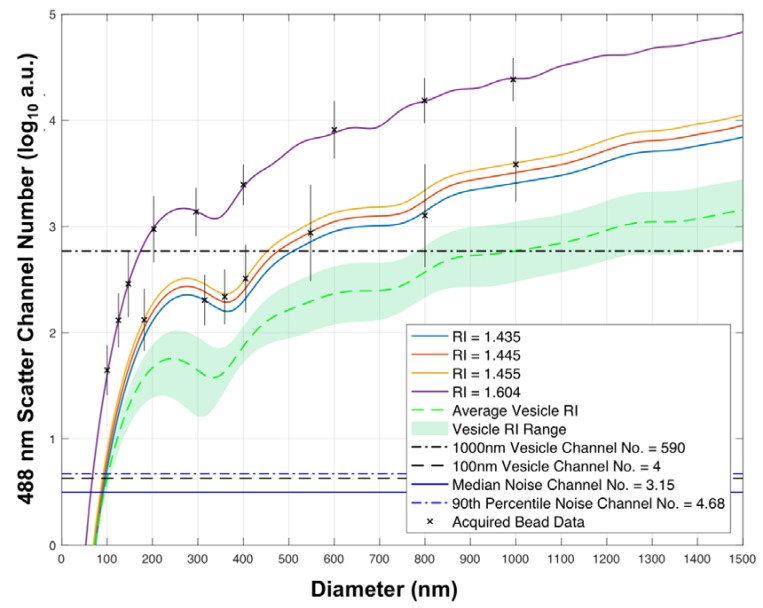
Scatter-diameter relationship of acquired vs. predicted polystyrene and silica particles on Astrios EQ. Predicted scattering cross-sectional units were normalized to flow cytometer arbitrary unit data using a single normalization factor. Error bars show the range of the acquired bead scattering in arbitrary units, with crosses indicating median acquired scattering of each bead.

**Figure 2:**
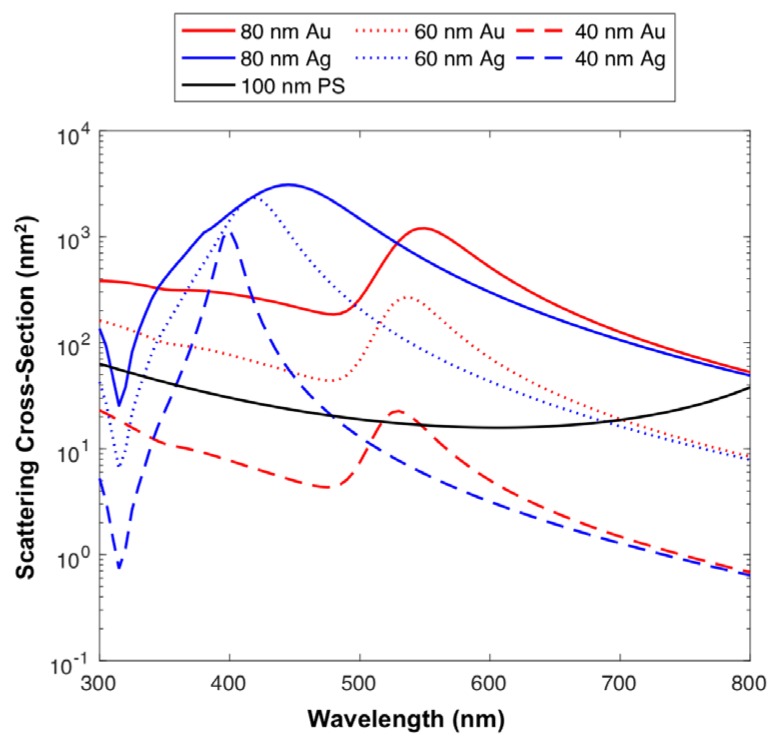
Modelled scattering cross-sections assuming Astrios EQ collection optics. Displayed are the scattering cross-sections of 40 nm, 60 nm, 80 nm Au (red) and Ag (blue) spheres and 100 nm polystyrene spheres (black).

Using previously developed methods of flow cytometer particle scatter modelling, Figure 1 combined with reference refractive index and extinction coefficients from the literature, it is possible to use these results, so as to predict which particle diameters and compositions may be detectable. Here, the side scatter collection optics of the Astrios EQ cytometer have been modelled to determine the relative detected scattering power of Au and Ag nanoparticles across the UV-visible spectrum, Figure 2. Other compositions (platinum, titanium dioxide, iron oxide, copper, lead, zirconium) were also modeled, Supplementary Figure S4.

The green lines represent the range of EV refractive indices (inner refractive index *n* = 1.38 ± 0.02, membrane refractive index *n* = 1.48 and a membrane diameter of 10 nm). The scattering power of 100 nm and 1000 nm EVs, assuming an average refractive index, are also shown (black lines). Instrument noise floor statistics are also displayed (blue lines).

Using 100 nm polystyrene (PS) beads as a cross-sectional scattering reference, due to them being detectable on a number of cFC platforms it can be seen that 20 nm silver nanoparticles illuminated at ~400 nm have a higher scattering cross-section than 100 nm PS particles, Figure 2. 40 nm Au particles surpass the scattering cross-section of 100 nm PS sphere at an illumination wavelength of ~532 nm, with 40 nm Ag particles having a high scattering cross-section with illumination wavelengths ranging from ~350–450 nm, Figure 2. 60 nm spheres of all compositions with the exception of cadmium selenide, iron oxide and titanium dioxide, have a higher scattering cross-section than 100 nm PS spheres across the majority of the UV-visible spectrum, before dropping in the red area (>700 nm) of the spectrum, Figure 2.

### 3.2. Modelled Particle Spectral Scattering versus Acquired Particle Spectral Scattering

**Figure 3:**
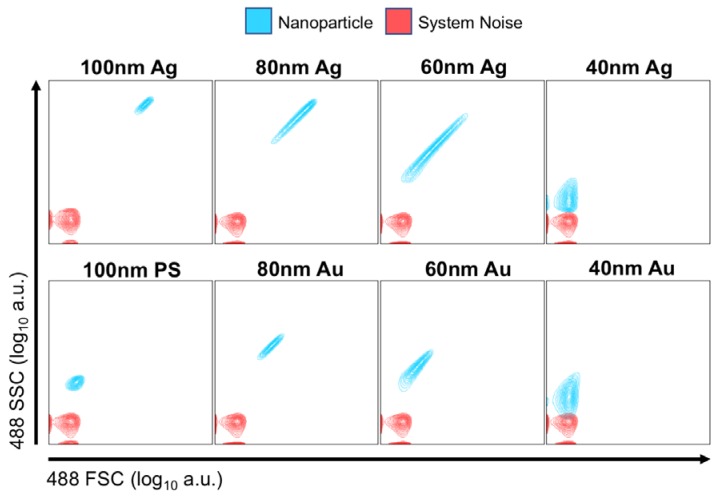
Comparison of the scattered optical power at 488 nm wavelength for gold (Au), silver (Ag) and polystyrene (PS) nanoparticles measured on Astrios EQ in the forward (FSC) and sideward (SSC) direction. Power levels are plotted on a logarithmic scale. The Astrios EQ instrument collects particle scattering at multiple wavelengths allowing quantification of system noise which is color coded. This is not easily done using the FACS Symphony and hence it is not color coded.

PS, Au and Ag nanoparticles were analyzed on the Astrios EQ (Figure 3) and FACS Symphony (Supplementary Figure S5) instrument to determine whether particles were detectable using the conventional scatter collection wavelength of 488 nm, Figure 3. Unstained 100 nm PS beads were resolvable on both instruments. 100, 80, 60 and 40 nm Ag particles were distinguishable from background on both instruments, with the 40 nm population being only partially resolved on the Astrios EQ and FACS Symphony from background noise. 20 nm Ag or Au particles were not resolved on either instrument. 80, 60 and 40 nm Au particles were resolved on both instruments, again with 40 nm Au being only partially resolved on each instrument. We note that these results are consistent with the simulations of Figure 2; at 488 nm wavelength for all sizes Au and Ag particles have similar scattering cross sections, with Ag having the slightly larger cross section as is also evident in the measured data.

**Figure 4:**
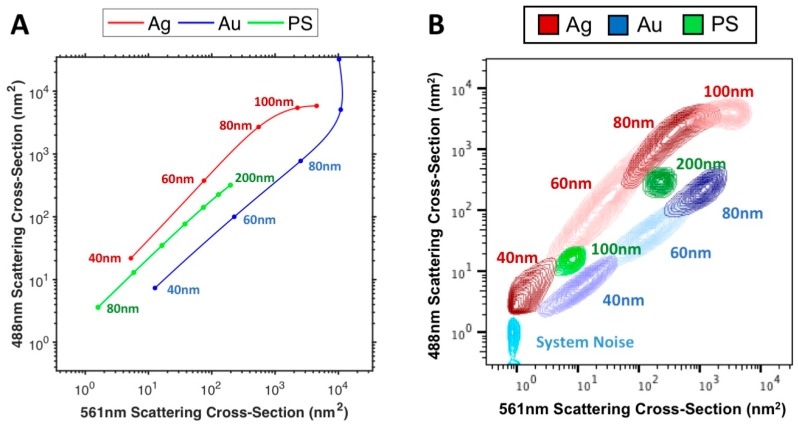
Multi-wavelength particle scattering models of the Astrios EQ (**A**) vs. acquired multi-wavelength particle scattering data on the Astrios EQ (**B**). Modelled data points are at diameters of 40 nm to 120 nm in 20 nm increments for Au and Ag and 80 nm to 200 nm in 20 nm increments for PS.

The PS, Au and Ag particles were then investigated using multi-wavelength modelling of the Astrios EQ, Figure 4A and comparing them to the acquired data from 488 and 561 nm SSC channels, Figure 4B. A comparison between modelled and acquired data at 405, 488, 561, 640 nm can be found in Supplementary Figure S6. Using the instrument background noise as a reference, 40 nm Ag particles were partially resolved on 561 nm, 488 nm and 405 nm SSC channels and unresolved on the 640 nm SSC channel. 40 nm Au particles were fully resolved on the 561 nm and 488 nm SSC channels, partially resolved on 640 nm SSC channel and unresolved on 405 nm SSC channel. 100 nm PS particles were fully resolved on 488 nm and 561 nm SSC channels, partially resolved on the 405 nm SSC channel and unresolved on the 640 nm SSC channel. All other particles were resolvable from instrument background noise on all SSC channels.

The relationship between Au, Ag and PS particle scattering on all channels was well maintained between models and acquired data, Figure 4A,B, Au particles show a linear increase in scattering between 488 nm and 561 nm SSC channels, with the PS particles appearing to also increase linearly but with increased 488 nm scattering than Au and finally Ag appearing to scatter linearly between 488 nm and 561 nm scattering channels before beginning to tail off in scattering on the 488 nm scattering channel between 60–80 nm but continue to increase in 561 nm scattering.

### 3.3. A Simple Method of Deconvulsion of Labelled EVs versus Unabled EVs

**Figure 5:**
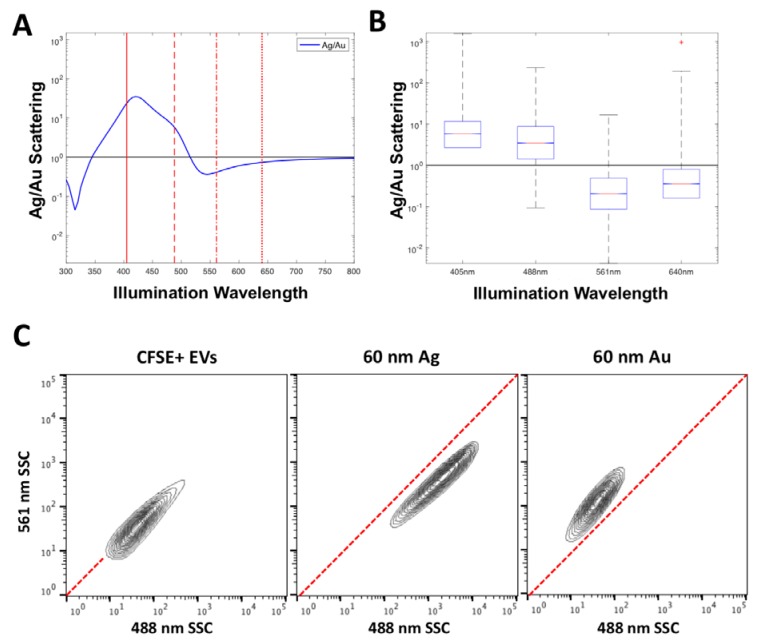
Simple spectral deconvulsion approaches to scattering labels. Illustrated is the predicted scattering cross-section ratio of 60 nm Ag to 60 nm Au particles (**A**) and acquired ratio of Ag to Au (**B**). Vertical red lines represent the particle scattering wavelengths (405, 488, 561, 640 nm) collected on the Astrios EQ instrument. The acquired scattering channel data for CFSE-stained EVs, 60 nm Ag and 60 nm Au particles on 488 nm and 561 nm SSC channels (**C**). The green area indicates a higher scattering power at 561 nm, while the red area indicates a higher scattering power at 488 nm. The red dotted line indicates an equal scattering power at 561 nm and 488 nm.

In order to implement the use of several labels at once, particles with unique spectral scattering properties would be required to provide a means of deconvolution. An example of two materials that show distinct spectral scattering properties are 60 nm Au and Ag, Figure 5A. By plotting the ratio of Ag to Au scattering, the wavelengths at which the most separation is likely to occur can be anticipated. It can be seen that 60 nm Ag particles will scatter more than 60 nm Au particles between the wavelengths of 350–510 nm, before the inverse occurs from 510 nm to 800 nm. This separation is confirmed in acquired data from the Astrios EQ which collects scatter at 405, 488, 561 and 640 nm, Figure 5B, the relative power of this comparison not possible in this form however, where the detector voltages affect the acquired channel number. It can however be seen that the acquired 60 nm Ag particles have a higher scattering intensity than 60 nm Au at 405 nm and 488 nm before inverting on the 561 nm and 640 nm wavelengths, as predicted from modelling.

The scattering properties of CFSE-stained EVs were then compared to those of 60 nm Au and 60 nm Ag particles, to show how their use as a label would enable distinction of epitope staining, Figure 5C. It can be seen that if CFSE-stained EVs were to be positively stained by an Ag NanoTag, their 488 nm and 561 nm SSC channel intensity would increase. If CFSE-stained EVs were to be positively stained by an Au NanoTag, mainly their 561 nm SSC channel intensity would increase. The characteristic channel intensities seen with Au and Ag NanoTags are distinct from one another and these differences provide a means for labeling two different epitopes or detecting two different EV-associated molecules in one assay. These results are expected based on the scattering properties of Au and Ag seen in Figure 2 and Figure 4B.

## 4. Discussion

We have shown that particles with diameters of 40, 60 and 80 nm, composed of Ag or Au, can be partially or fully resolved by Astrios EQ and FACS Symphony flow cytometers. However, these cytometers may be considered ‘high-end’ pieces of equipment and not all conventional flow cytometers are capable of detecting the 100 nm polystyrene spheres used as a reference standard in our work here. While we have demonstrated that our flow cytometers are capable of detecting 40 nm particles at multiple scattering detection wavelengths, an ideal instrument for analysis of multiple (>3) spectral scatter labels simultaneously would utilize a broad range of illumination wavelengths; this could be in the form of a supercontinuum white laser, as well as multiple scatter detection channels. While using just one or two labels is feasible using a conventional flow cytometer configuration with scattering collection filters.

Due to the limited surface area and therefore surface proteins on EVs, steric hindrance is likely a problem for current immunoglobulin labels, which are approximately 15 nm in diameter. While this is an issue, especially for an immunoglobulin labelled with a few fluorophores that are undetectable on current instruments, only one NanoTag need bind to an EV surface epitope for it to be detectable. A 100 nm EV has the capacity to bind ~20 loosely packed 15 nm spherical particles. Concentrations of NanoTags to EVs could therefore be far lower, not saturating EV surfaces, thereby allowing multiple NanoTags targeted to different proteins to bind. Another important factor when considering the use of nanoparticles as labels is their conjugation. Many conventional nanoparticle labels, such as Qdots, result in polyvalent labels. To ensure single NanoTag binding to EVs the development of monovalent labels would be instrumental. Monovalent labelling of Qdots has previously been demonstrating by wrapping DNA around the nanoparticle [6]. This method leaves a single functionalized end group that can be used to bind antibodies, aptamers, and so forth. A priority of future work to develop NanoTags as a viable labelling method would need to focus on creating a monovalent labelling methodology such as this.

As single NanoTags are capable of being detected without being bound to a targeted particle, such as an EV, a method of distinguishing labeled from unlabeled EVs are is required. One approach is to fluorescently label all EVs with an intercalating membrane dye, or non-specific stain such as CFSE [16]. It would then be possible to identify a shift in a NanoTag’s fluorescence when bound to a labelled EV, to determine whether it has labelled an EV or not. Alternatively, if a specific type of EVs were of interest for example, phosphatidylserine positive, it would be possible to make a NanoTag specific for the population of interest and use a second NanoTag targeted at an EV subset for example, CD9 positive. A unique scattering distribution would then occur for EVs that had two NanoTags, each with distinct scattering properties, bound to them.

In summary, single 40, 60 and 80 nm Au and Ag particles are not only detectable using conventional forms of flow cytometry but are also uniquely distinguishable from one another and fluorescently-labelled EVs, by using their spectral scattering properties. It is feasible that particles of such diameter and composition could be used as polyvalent or even monovalent detection labels, using currently available labelling methods, as means for single-epitope detection and implemented as single-epitope detection tags.

Our proposal for Molecular NanoTag as labels leverages the light scattering properties of high refractive index or highly plasmonic nano-materials, thereby providing a signal high enough for single molecules detection using currently available flow methodologies and may therefore be useful as labels for low expression, low scatter targets, such as EVs.

There are two main applications where Molecular NanoTags fill an existing gap in available detection methods. First, there are no methods currently available whereby a clinical laboratory is able to take a blood sample at and determine how many EVs are positive for a specific tumor marker per unit volume of blood. Rather current methods are only able to bind EVs to multi-micron sized beads and use detection antibodies to detect the EVs bound to the larger beads. Since individual Molecular NanoTags are labels that can be resolved individually, the detection of EVs with as few as one Molecular Nanotag-labelled receptor is feasible. Second, assembly of the Molecular NanoTags in a manner that ensures that the labels are monovalent enables enumeration of the number of labeled molecules. Such molecular enumeration is a significant advancement beyond the enumeration capabilities of current flow cytometric labels and instruments.

## 5. Patents

Jones JC, et al., “Molecular NanoTags”. Provisional U.S. Patent Application No. 62/411,324; PCT Patent Application No. PCT/US2017/057928 filed 23 October 2017.

## Supplementary Materials

The following are available online at http://www.mdpi.com/1424-8220/18/8/2461/s1, Figure S1: Refractive index (A) and extinction coefficient (B) of zirconium, titanium dioxide, platinum, lead, iron oxide, copper, cadmium selenide, gold, and silver at wavelengths between 300–800 nm.; Figure S2: Serial dilutions of 40 nm (A,B), 60 nm (C,D), and 80 nm (E,F) Au nanoparticles.; Figure S3: Modelling data of each detection wavelength.; Figure S4: Modelled scattering cross-sections assuming Astrios EQ collection optics.; Figure S5: Comparison of the scattered optical power at 488 nm wavelength for gold, silver, and polystyrene nanoparticles measured on Astrios EQ and FACS Symphony flow cytometers in the forward (FSC) and sideward (SSC) direction. Figure S6: Multi-wavelength particle scattering models of the Astrios EQ vs. acquired multi-wavelength particle scattering data on the Astrios EQ.
