# Supplementary material for: Prospective Use of High-Refractive Index Materials for Single Molecule Detection in Flow Cytometry
Source: Sensors (Basel). 2018 Aug 1;18(8):2461. doi: 10.3390/s18082461 (PMC6111846; doi:10.3390/s18082461)
Supplement: Supplementary file 1 [file sensors-18-02461-s001.pdf]

sensors

MDPI

Supplementary Materials: Prospective Use of High-Refractive Index Materials for Single Molecule Detection in Flow Cytometry

Joshua A. Welsh [1], Julia Kepley [1], Ariel Rosner [1], Peter Horak [2], Jay A. Berzofsky [1] and Jennifer C. Jones [1,*]

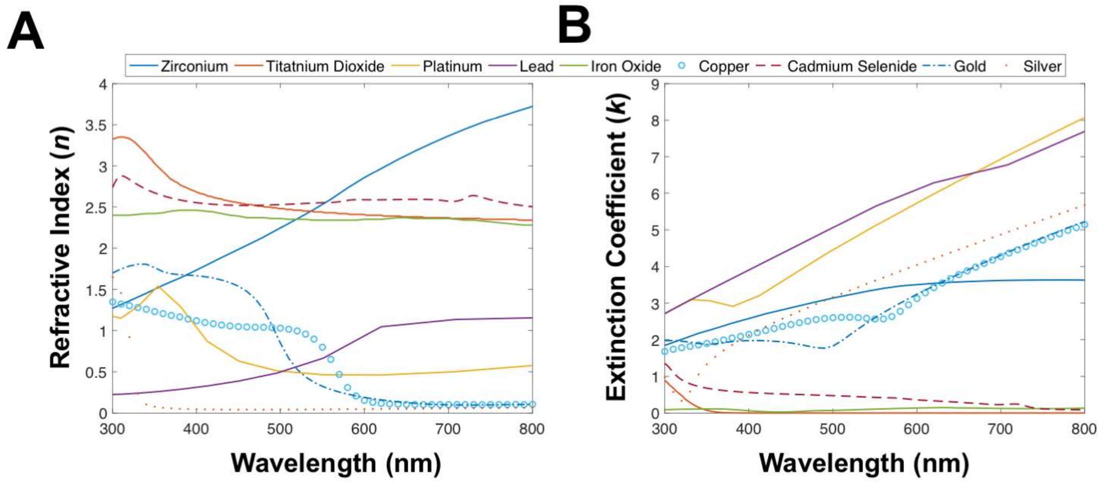

Figure S1. Refractive index (**A**) and extinction coefficient (**B**) of zirconium, titanium dioxide, platinum, lead, iron oxide, copper, cadmium selenide, gold, and silver at wavelengths between 300–800 nm.

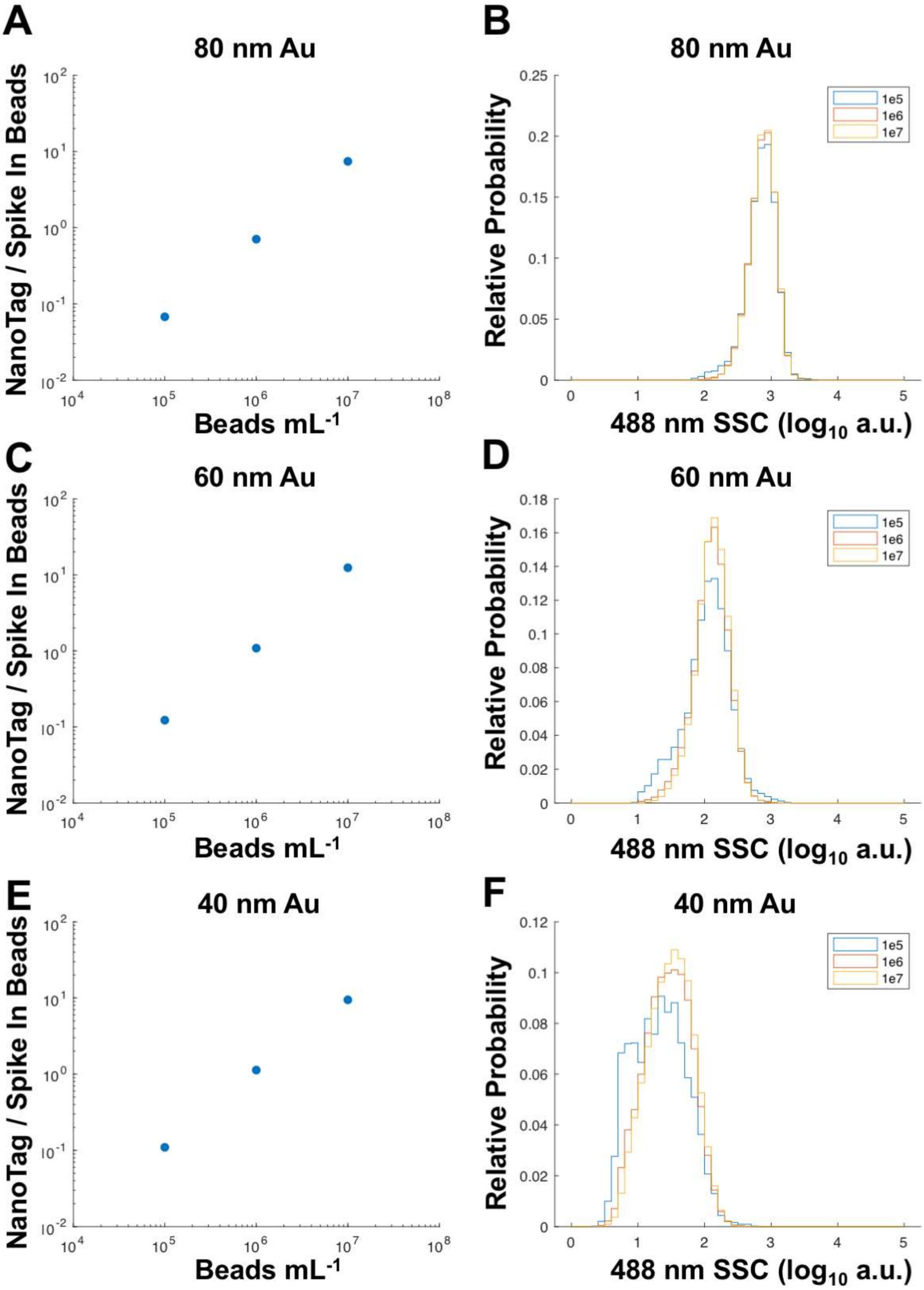

Figure S2. Serial dilutions of 40 nm (**A,B**), 60 nm (**C,D**), and 80 nm (**E,F**) Au nanoparticles. Au nanoparticles were diluted to 1e7, 1e6, and 1e5 particles mL^{-1} with a spike in of 200nm fluorescent polystyrene beads at 1e7 particles mL^{-1}. The ratio of detected Au to 200nm polystyrene was plotted against the prepared concentration of Au. It can be seen the ratio of diluted Au to spike beads vs. the prepared Au concentration obtains a linear decrease. This is indicative of single-particle detection. Furthermore, there is no appreciable difference in particle scatter signal at varying dilutions, further indicative of single particle detection.

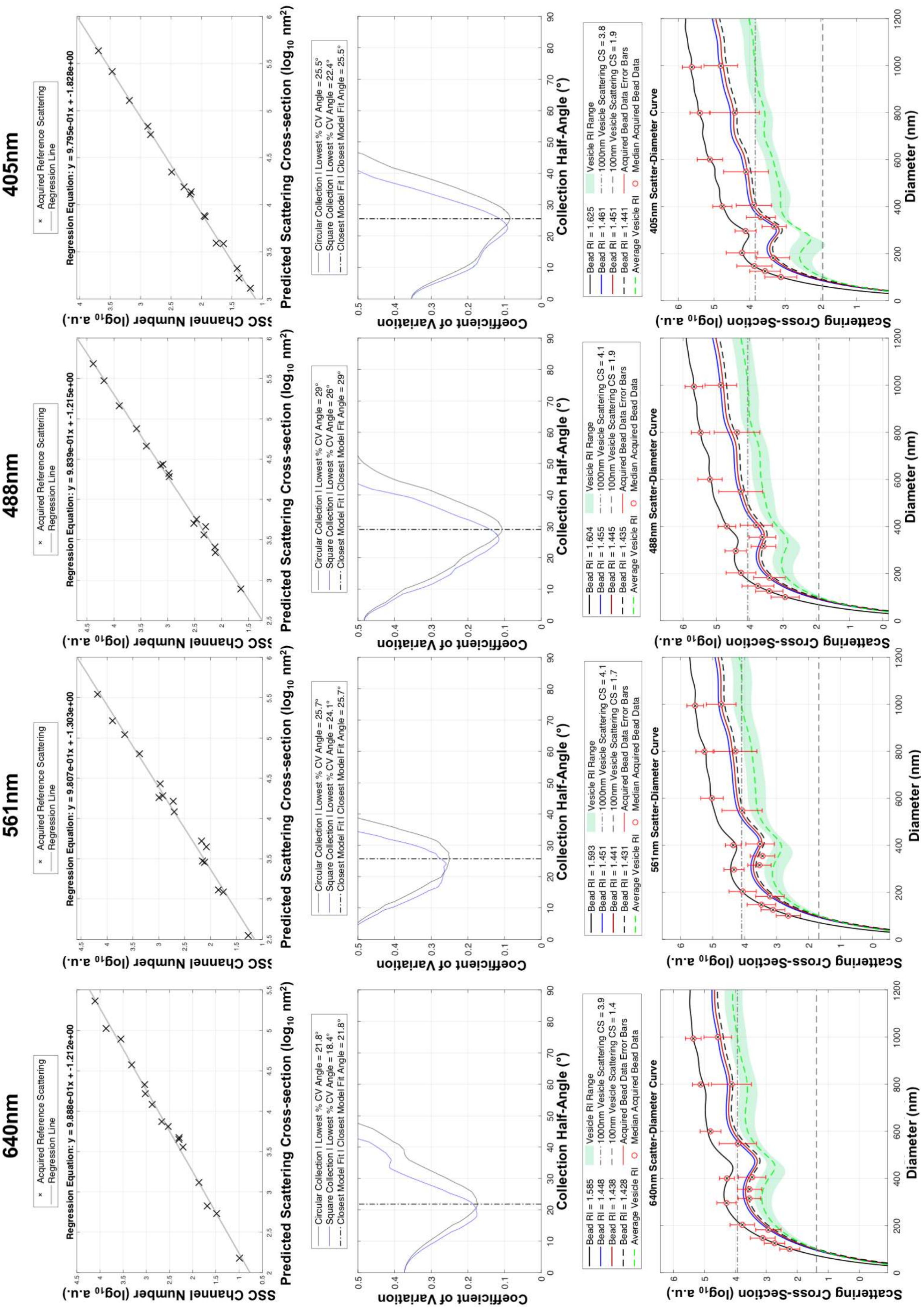

Figure S3. Modelling data of each detection wavelength.

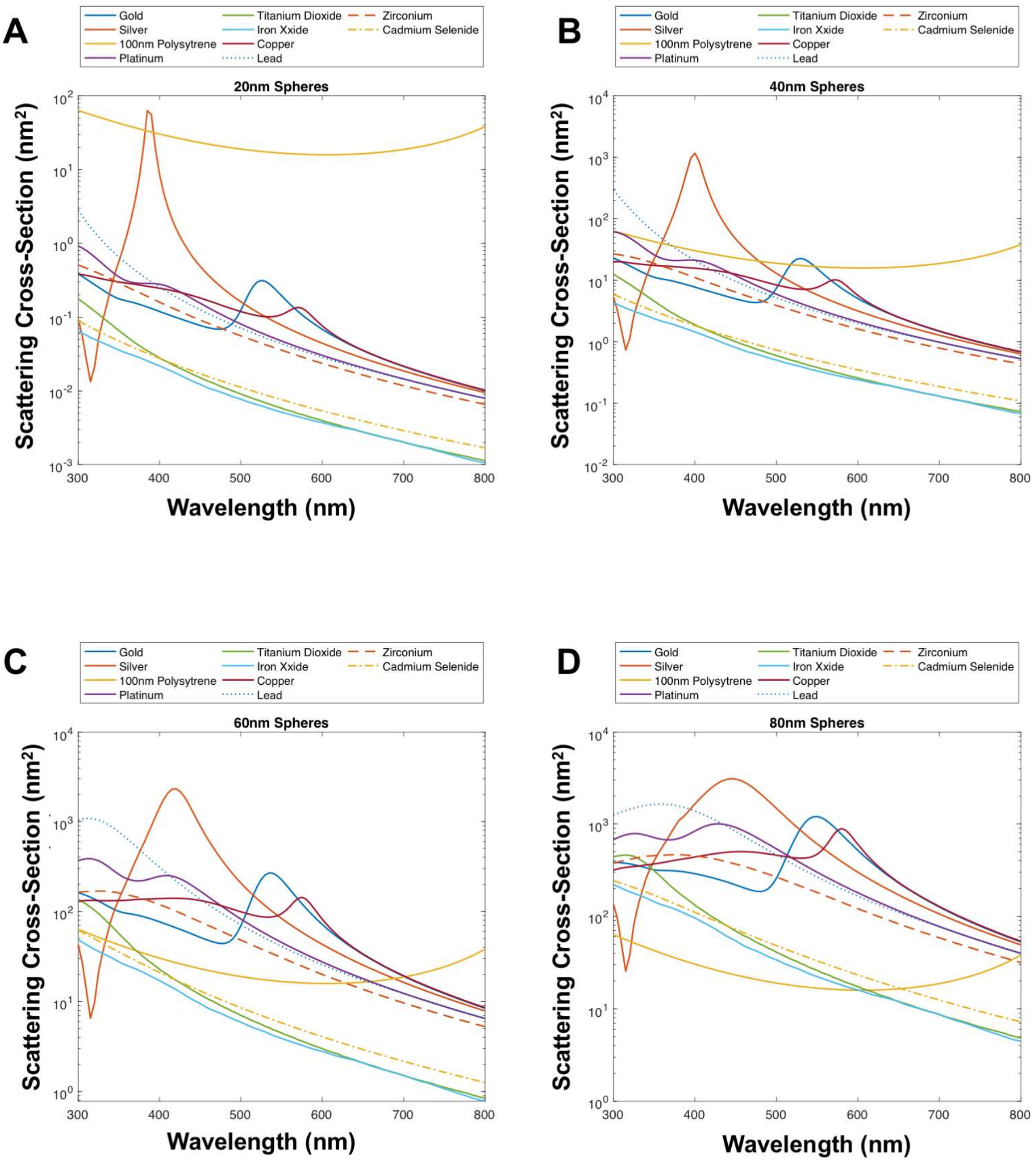

Figure S4. Modelled scattering cross-sections assuming Astrios EQ collection optics. Displayed are the scattering cross-sections of 20 nm (**A**), 40 nm (**B**), 60 nm (**C**), 80 nm (**D**), of particle compositions; zinc, titanium dioxide, platinum, lead, iron oxide, copper, cadmium selenide, gold, and silver.

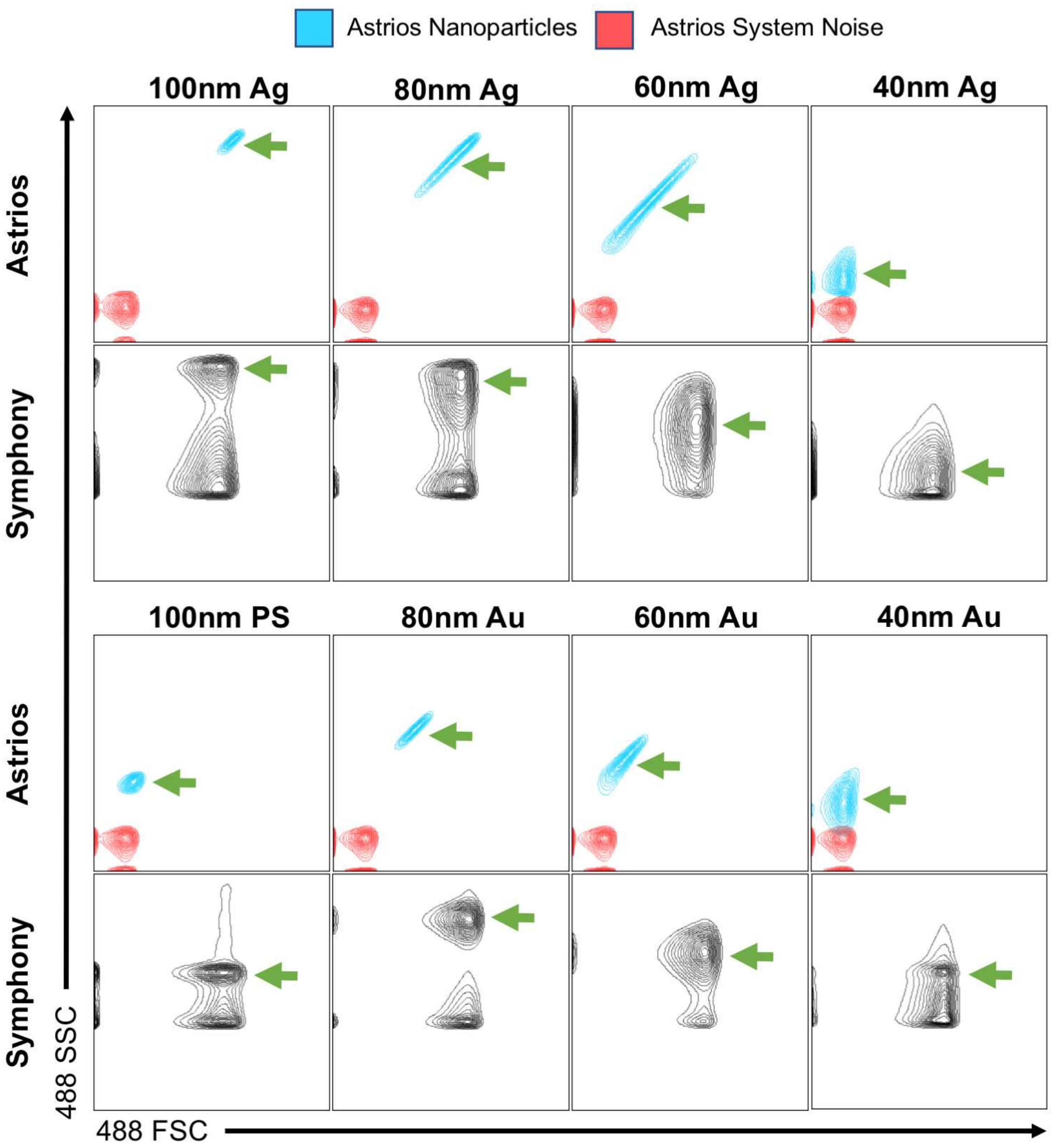

Figure S5. Comparison of the scattered optical power at 488 nm wavelength for gold, silver, and polystyrene nanoparticles measured on Astrios EQ and FACS Symphony flow cytometers in the forward (FSC) and sideward (SSC) direction. Power levels are plotted on a logarithmic scale. Green arrows indicate nanoparticles. The Astrios EQ instrument collects particle scattering at multiple wavelengths, allowing quantification of system noise, which is color coded. The discrimination of system noise with the FACS Symphony is not easily done, and, hence, it is not color coded.

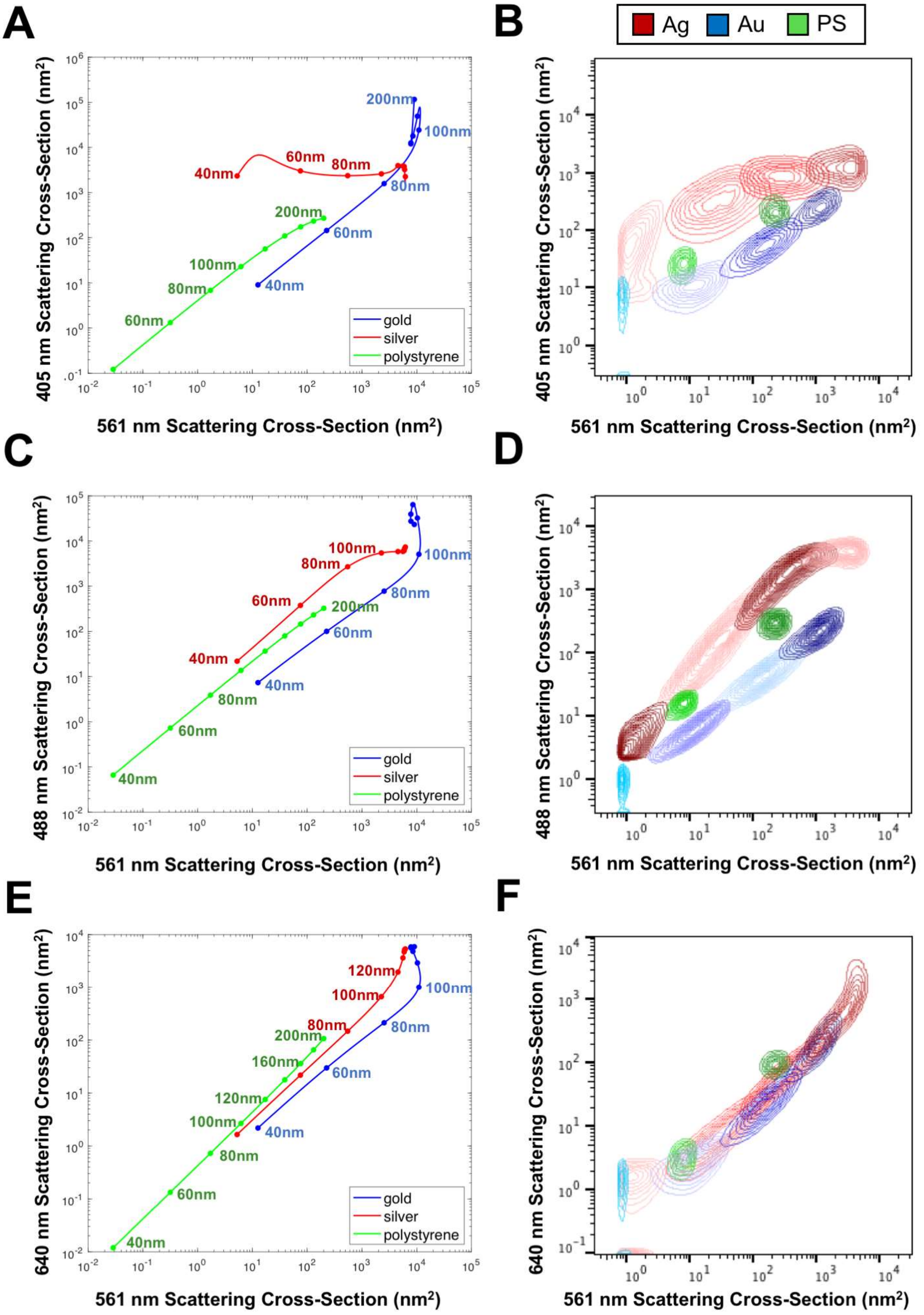

Figure S6. Multi-wavelength particle scattering models of the Astrios EQ (**A**, **C**, **E**) vs. acquired multi-wavelength particle scattering data on the Astrios EQ (**B**, **D**, **F**). Modelled data points are at diameters of 40nm to 200nm in 20nm increments.
